# Trade-offs between remembering and online evaluation in retrospective evaluation

**DOI:** 10.3758/s13423-026-02970-z

**Published:** 2026-07-31

**Authors:** Alice Mason, Geoff Ward, Gordon D. A. Brown, Simon Farrell

**Affiliations:** 1 University of Bath, Claverton Down, Bath, BA2 7AY UK; 2 University of Essex, Wivenhoe Park, Colchester, CO4 3SQ UK; 3 University of Warwick, Coventry, CV4 7AL UK; 4 School of Psychological Science, University of Western Australia, 35 Stirling Highway, Crawley, WA 6009 Australia

**Keywords:** Memory, Evaluation, Dual-task, Willingness-to-pay

## Abstract

This paper examines the trade-off between remembering and online evaluation in retrospective judgments. In two experiments, we compared conditions where participants performed only one task (evaluation or recall) versus both tasks, with task expectancy manipulated through pre-cueing or post-cueing. Participants (*N*=139 and *N*=262) viewed sequences of numbers representing monetary values and were asked to either recall the numbers or evaluate the sequence through willingness-to-pay judgments. Results showed that when both tasks were potential (Experiment 1) or actual (Experiment 2) candidates to be cued, one or the other task showed a decrement in performance, indicative of some dual-task cost. Strikingly, simulations revealed that participants would have performed better had they focused solely on memory encoding during presentation rather than attempting dual-task processing. These findings suggest that people do not optimally allocate cognitive resources between competing processes during retrospective evaluation, and may attempt to deploy multiple specialized strategies even where a memory-based strategy would be effective overall.

**Supplementary Information:**

The online version contains supplementary material available at https://doi.org/10.3758/s13423-026-02970-z.

We often need to make retrospective judgments about experiences, either to communicate our impression to others, or to provide some basis for choosing between options. While the best summative representation of an experience as a whole would usually be the sum of the individual elements of the experience, people’s judgments sometimes indicate other strategies are at play. For example, retrospective evaluations are characterized by duration neglect: people’s judgments imply that they average rather than sum across elements of an experience (e.g., Fredrickson & Kahneman, 1993). One key and diagnostic phenomenon is that people often overweight the most recent elements, and give more weighting to those elements that are most positive or negative in a sequence (i.e., the sequence peak; Aldrovandi et al., 2015; Fredrickson, 2000; Langer et al., 2005). This pattern of peak and end effects has been found in such diverse situations as human–computer interactions (Cockburn et al., 2017), payment sequences (Langer et al., 2005), information about carbon footprint (Sörqvist et al., 2024), and painful medical procedures (Redelmeier et al., 2003).

The pattern of overweighting of distinctive and recent elements of an experience accords well with the better recall of recent and distinctive items in the memory literature (Hunt, 1995; Murdock, 1962), such that some researchers have suggested that the pattern of overweighting in retrospective evaluations can be explained by memory biases (Aldrovandi et al., 2015; Montgomery & Unnava, 2009; Sörqvist et al., 2024). Montgomery and Unnava (2009) found that inserting a delay after the sequence shifted the memory serial position from recency to primacy – as is typically found in delayed recall studies (Howard & Kahana, 1999) – and that primacy elements were similarly overweighted in retrospective judgments. Montgomery and Unnava (2009) and others (e.g., Aldrovandi et al., 2015) also found that when both memory and evaluations were obtained for individual sequences, recall was a better predictor of the evaluation than an estimate formed from all items in the sequence.

While such demonstrations suggest that memory is involved in evaluation, Hastie and Park (1986) noted that in any particular situation, evaluation could either be based on memory or result from an online evaluation of the sequence while it was being experienced or presented (Hogarth & Einhorn, 1992). Hastie and Park (1986) suggested that memory is likely only to be involved when online evaluation is disfavored or not possible; for example, when the requirement to evaluate is unexpected. Aldrovandi et al. (2015) showed that reliance on memory is evident even when evaluation is expected, and this is in line with evidence from related judgment and learning tasks showing a role for episodic memory (e.g., Bornstein et al., 2017; Plonsky et al., 2015; Duncan & Shohamy, 2016), even in cases where a reliance on memory may be maladaptive (Noh et al., 2023).

In recent work, Mason et al. (2024) used model comparison to provide a detailed examination of the relative role of memory and online evaluation in judgments of monetary sequences, and how those differ between individuals. Mason et al. presented participants with sequences of numbers representing currency values (cf., Langer et al., 2005) and asked participants to both evaluate each sequence and freely recall values from the sequence. By fitting a variety of models, Mason et al. were able to quantify the evidence that participants were relying on memory, on averaging, or on a mixture of both. Mason et al. (2024) found that only a small number of participants relied purely on recall to form evaluations. Most participants appeared either to use an online strategy that effectively calculated the average of the sequence, or to use a combination of online processing *and* memory. As well as showing that memory need not be ubiquitous as a strategy for retrospective evaluation – even in cases where recall is required (Aldrovandi et al., 2015) – Mason et al.’s results suggest a shift in focus to ask about the relative mixture of evaluation and memory, how that varies across people, and what determines that mixture for any particular situation.

How do people choose between different strategies, and how is a mixture of strategies coordinated? One suggestion relating to the role of memory in control of behavior more generally is that the brain should optimally tailor the use of different systems according to the challenges of the task at hand, so as to minimize costs and maximize reward (e.g., Nicholas et al., 2022; Lengyel & Dayan, 2007; Poldrack et al., 2001). In cases where people could rely on episodic or more incremental reinforcement learning, Nicholas et al. (2022) found that episodic memory was relied on more in cases where the environment was more volatile, and thus where incremental learning would provide less accurate predictions. In a task where “reminders” using incidental context have been found to prompt the use of episodic memory (Bornstein et al., 2017; Noh et al., 2023) found that this effect was related to the precision of memory, with memory sampling having a greater effect on choice for those cases where memories were more precise.

In the case of a mixture, one model suggested by Hastie and Park (1986) in the case of retrospective evaluation is that online evaluation and memory encoding occur concurrently, with information being encoded into long-term memory and independently being fed into the process of online evaluation. While Hastie and Park (1986) suggested that these processes occur independently, it is possible that both memory encoding (e.g., Craik et al., 1996) and online evaluation (Hastie & Park, 1986) will be demanding on cognitive processing. In such situations, we might expect a trade-off between the two processes during list presentation, raising the question of how participants manage that trade-off given the consequences for performance.

To answer this question, we compared conditions where participants complete only one task (either evaluation or recall) to the case where people complete both. If people are relying on both online evaluation and memory, this should effectively represent a dual-task challenge at encoding, as examined in studies of divided attention (e.g., Baddeley et al., 1969; Craik et al., 1996). Par th sequences of seven values, and we manipulated both task (recall versus evaluation) and task expectancy. In the sole task condition, participants were pre-cued to complete either the evaluation or the free recall on all trials. In the post-cue condition, participants were retrospectively cued on a trial-by-trial basis to complete either evaluation or recall (Experiment 1) or were cued to complete both in an unpredictable order (Experiment 2). Both tasks were incentivized in a way that allowed us to examine trade-offs between memory and evaluation in a common currency.

## Experiment 1

### Method

#### Participants

We collected data from 150 participants as per our preregistration. Five participants were excluded for not completing at least 80% of the experiment and six were excluded for not completing 80% of the evaluation trials. A total of 11 participants were excluded in line with the preregistration, and 135 were included in the analysis (*N* = 44, 50, and 41 in the pre-cued WTP, pre-cued memory, and post-cued conditions, respectively). Participants were recruited via Prolific Academic to participate in the experiment online. To be eligible to take part in the experiments participants needed to be aged 18–65, have English as their first language (self-reported), be a resident of the UK, USA, Ireland, Australia, New Zealand or Canada, and have a Prolific Academic approval rating of at least 90%. Participants were reimbursed for their time according to the standard rates on Prolific Academic at the time the experiment was conducted, and could earn an additional performance-related bonus.

#### Design

The experiment adopted a between-subjects design, with different participants participating in the three task expectancy conditions: memory only, evaluation only, or both. For analyses, this collapses to a two-level variable of pre-cued vs post-cued, where we compare a task in the “both” condition to the corresponding task completed in isolation (that is, memory-only or evaluation-only).

#### Materials

On each of 32 trials, participants were presented with a sequence of seven two-digit numbers. The sequences were uniformly sampled, and the minimum and maximum values varied across each list but were always between 11 and 99. When generating the sequences, we made within-sequence variability large relative to between-sequence variability to make it obvious that sampling more numbers would improve accurate estimation of the mean. To achieve this, the mean of each sequence was randomly sampled from the numbers 40 to 70 (a uniform distribution). We then calculated the highest and lowest value for the distribution of each sequence by adding or subtracting 24 from the mean (i.e., each sequence distribution had a range of 58 but different minimum and maximum values). Finally, seven numbers were uniformly sampled from this sequence distribution.

#### Procedure

Participants were informed that they would be presented with sequences of two-digit numbers that represented amounts in a fictitious currency, Galactic Credits (GC). Accordingly, each sequence had a currency value that was simply the average of the numbers in that sequence.

Each trial began with the sequential presentation of the seven numbers. Each number was presented one at a time for 1500 ms, with an inter-stimulus interval of 1000 ms. Following presentation of the numbers, participants were cued to recall the sequence, evaluate the sequence, or both.

If a recall cue appeared, participants had 15 s to recall as many items as possible in any order. Participants were instructed to type their responses, pressing Enter after each item, into a response box at the center of the screen. The screen was cleared each time the participant pressed Enter so that previous responses were not visible. We chose a short recall period of 15 s; Aldrovandi and Heussen (2011) suggested that when a longer period of 2 min is used, participants engage in exhaustive recall, whereas we were interested in the immediately accessible items that would plausibly contribute to evaluation in this setting and in everyday life.

If the cue to evaluate appeared, participants took part in an incentive-compatible auction (Becker et al., 1964). Participants were informed (in initial experiment instructions) that they were taking part in a willingness-to-pay (WTP) task. For each trial, and therefore for each bid, participants were given an endowment of 100 Galactic Credits. Participants placed a bid for each sequence (also in Galactic Credits), and a selling price was randomly drawn from a uniform distribution of prices (the range used in the experiment). If the bid was below the selling price, the participant did not purchase the item and kept only the endowment. If the bid was above the randomly drawn selling price, then the participant automatically used their endowment to buy the sequence at the selling price, and kept the remainder of the endowment. In this latter case, the participants earning was then the remainder of the endowment, plus the currency value (the average) of the sequence that had been purchased. The optimal strategy – placing a bid equal to the estimated true value of the sequence – was explained to participants. The use of an auction to measure willingness to pay followed Mason et al. (2024), and is a common technique to elicit true valuations in behavioral economics (Becker et al., 1964; Lusk & Shogren, 2007) and neuroeconomics (Newton-Fenner et al., 2023).

Participants in the condition where both tasks were completed for each sequence knew they would be asked to complete one of the two tasks; the task cued for varied pseudo-randomly in such a way that each task was cued for half the trials. Both the free recall task and the evaluation task were incentivized. In the recall task, participants were awarded 10 Galactic Credits for each correctly recalled number. The evaluation task is incentive-compatible as only the participant’s bid determines whether or not they buy the item, and the greatest payoff would be obtained by a participant bidding what they believed to be the true value of the sequence.

At the end of the experiment, the computer program randomly selected two trials to be paid out based on the Galactic Credits earned on that trial (using a conversion rate of 200 Galactic Credits = £1). The conversion rate was revealed to participants at the end of the experiment; in the initial instructions, it was indicated that the bonus would likely lie between £0 and £2.

**Fig. 1:**
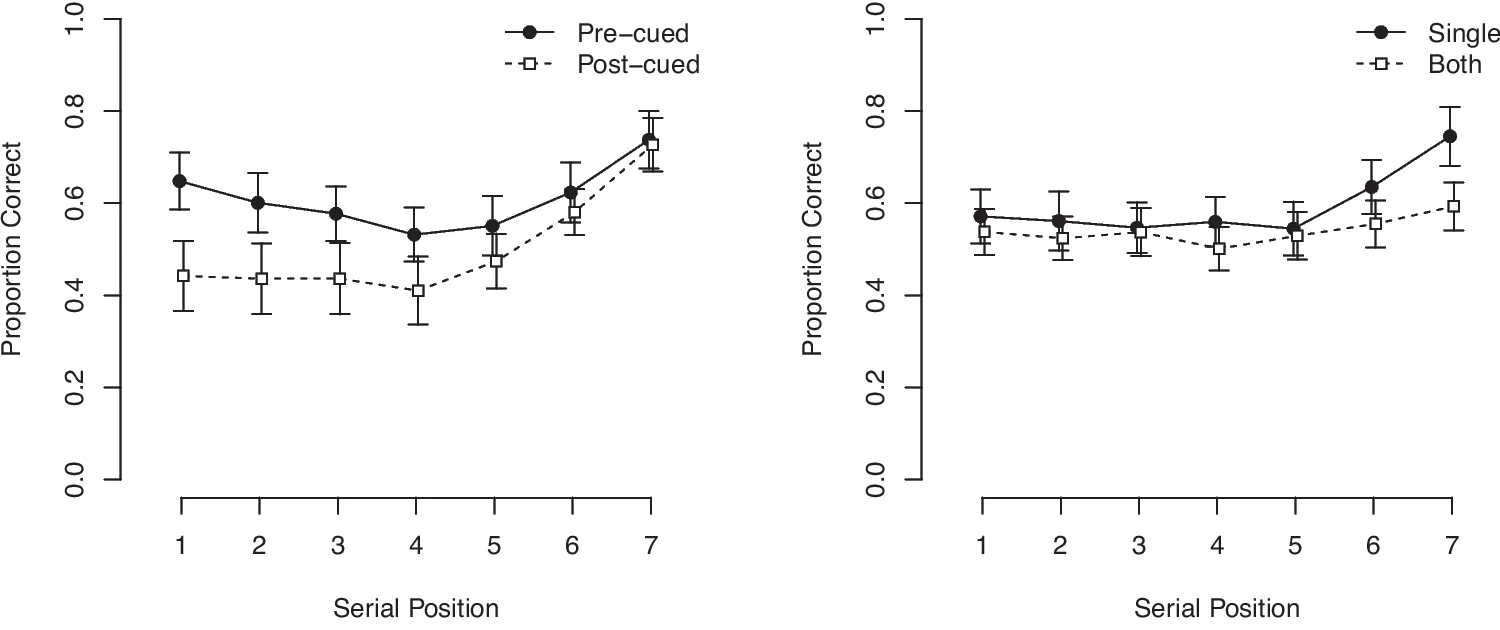
Recall accuracy as a function of serial position for Experiment 1 (*left panel*) and Experiment 2 (*right panel*)

#### Data analysis

The inferential framework used was Bayesian estimation and model comparison. For the analyses where mean recall or WTP were compared across conditions, we used the BayesFactor package (Morey & Rouder, 2014) to estimate Bayes factors. For these Bayes factor ANOVA analyses, the scale of the effect size for fixed effects (*r* scale) was set to .707, labeled the "medium" prior in the package. Where we are testing multiple effects, we use the "top" method to estimate Bayes factors, otherwise, the default is to compare against a null model that only has a subject-specific intercept. For *t* tests, the analyses used an uninformative Jeffrey’s prior on the variance, and a standard Cauchy prior of $\sqrt{2}/2$ on the *r* scale value (see Rouder et al., 2016).

The value of a Bayes factor quantifies the strength of evidence in favor of one model, with respect to another, given the data obtained. It informs us how much our prior beliefs in the models should shift in response to the data obtained.

The pre-registration can be found at https://osf.io/ean6u/ (for Experiment 2, see https://osf.io/cvpmy/registrations). The following departures from preregistration are noted. For the analysis of memory performance, a simpler Bayesian repeated-measures ANOVA is used in preference to a mixed-effects logistic regression. Joint estimation of memory and evaluations was not possible as both memory and evaluations were not collected on the same trials, and this was addressed instead in the pre-registered Experiment 2. The choice to terminate data collection at 150 participants was based on the main effect of condition in the memory accuracy data, and response times in the WTP task; lag-CRP was not considered in the decision to terminate as we subsequently deemed it less relevant to the main question of interest.

### Results

It was predicted that if there is a trade-off between memory and sequential updating at encoding, memory would be less accurate in trials where the task is post-cued. Figure 1 shows the effects of the task expectation on recall performance in the memory task, with a detrimental effect of post-cueing the task on performance. This was confirmed with a Bayesian ANOVA, which found the best-supported model was the one that included all effects (including the interaction). This model was well-supported (*BF*=14.99) over the next best-fitting model, which contained the main effects of condition and serial position. Of primary relevance is the main effect of condition, where recall accuracy was substantially better in the pre-cued condition (*M*=.61) compared to the post-cued condition (*M*=.50), supported by a Bayes factor of 27.35 against the null model. This indicates that expectation of potentially having to perform evaluation on the list led to a cost at encoding.

It was also predicted that condition would affect the serial position functions for the WTP task. The WTP value for each trial was predicted from the presented values according to their serial position (1–7) using a Bayesian linear mixed effects model, with a random effect for intercept. Figure 3 shows the parameter estimates arising from fitting the pre-cued and post-cued conditions separately (left panel); the regression estimates indicate the weighting given to each value in determining the observed WTP value on each trial. While Fig. 3 suggests that the post-cued condition produces more recency in the weighting function, model comparison with a baseline model including only an effect of serial position supported that simpler model (BF=3.48e06) and implied only trivial differences in the weighting functions between the conditions.

**Fig. 2:**
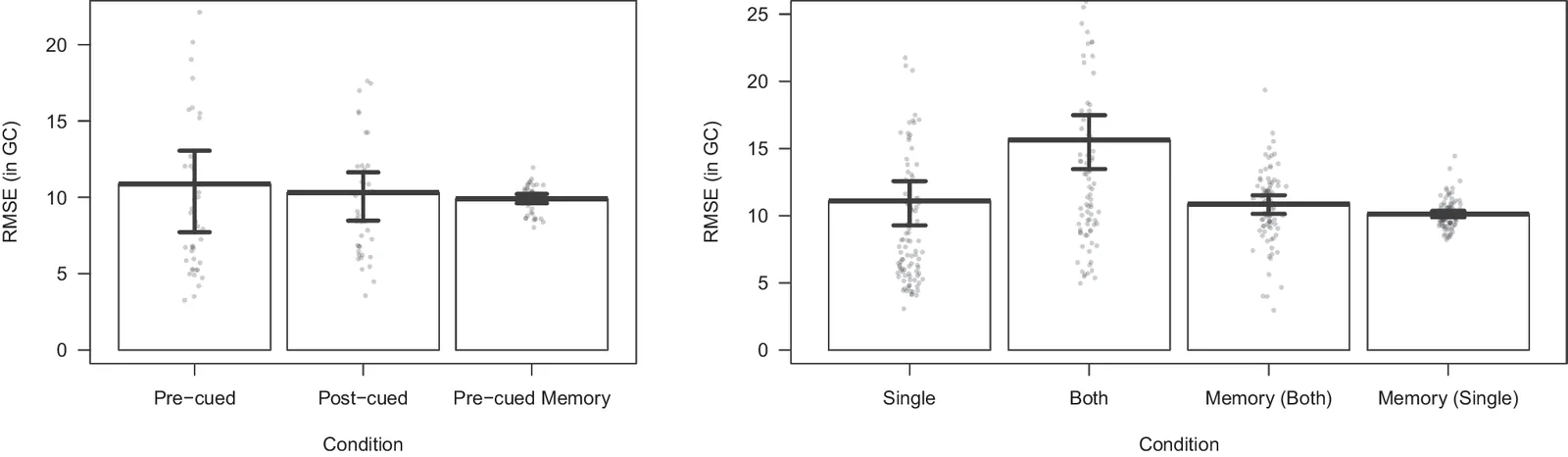
Effects of task expectancy on evaluation performance (root mean square error) in Experiment 1 (*left panel*) and Experiment 2 (*right panel*). Note: The “pre-cued memory” performance (*left panel*) and “memory (single)” (*right panel*) and is predicted evaluation performance based on recall of those participants in the pre-cued condition. “Memory (both)” (*right panel*) is evaluation predicted from memory performance for those same participants. See text for details

A similar implication arises from examination of accuracy in the WTP task. The left bars in the left panel of Fig. 2 show evaluation (WTP) performance as a function of task expectancy. Reinforcing the visual pattern shown in the figure, the Bayes factor of $BF_{01} = 0.19$ from a between-subjects *t* test revealed some evidence against an effect of task expectancy (i.e., in favor the null hypothesis of no difference).

We pre-registered an additional analysis regarding the speed of WTP evaluations, with the idea that participants might be faster to make evaluations if the enactment of evaluation is affected by a potential recall requirement. There is substantial evidence that participants were faster to make evaluations in the sole task condition (*M*=3458 ms) than in the post-cued condition (*M*=5369 ms), $BF_{10} = 36,035$. While this result is in line with a slower memory-based process, some of the effect can be attributed to initiation of response prior to cue, consistent with an analogous slowing observed in initial response times on the recall task (*M*=2661 ms vs *M*=3199 ms, $BF_{10} = 21.97$).

If participants are trading off memory encoding against online updating, we should be able to quantify this trade-off as both memory and evaluation can be measured on the same scale. In particular, we can ask about the consequences of turning the task into a dual-task – and attempting to jointly carry out online updating and memory encoding – for performance in terms of earnings on the WTP task. In other words, did the dual-task cost participants appear to have imposed on themselves result in increased earnings overall, compared to the alternative strategy of simply performing memory encoding, and then performing evaluation based solely on recall?

To answer this, we conducted an exploratory analysis across experiments to examine how accurate participants would have been in the evaluation task if they had based their WTP estimates on memory recall where memory encoding had not been impacted by processing apparently dedicated to online evaluation. For each participant in the post-cued condition who performed evaluation, we selected a random yoked participant from the pre-cued recall condition. For each yoked participant, we used their (correct) recalls (which serial positions were correctly recalled from on each trial) to simulate evaluation performance based on the values present on each trial for the participant in the post-cued evaluation condition, and averaged the RMSE values across simulated trials. The bar “Pre-cued Memory” in Fig. 2 shows that had participants used this pure memory-based strategy – encoding items as if for a memory task, and then evaluating based on recalled items – they would have performed as well as they did in the two actual evaluation conditions. Together with the detrimental impact of post-cueing memory seen in Fig. 1, this implies that participants were apparently choosing to turn the post-cued task into a dual task (as reflected by the cost of the post-cueing to memory accuracy) with no benefit to WTP accuracy, when relying just on a memory-based strategy (and thus not incurring a cost to memory performance) would also have benefited WTP performance. The cost of this apparently non-optimal approach can be quantified: participants in the post-cued condition scored an average of 39.86 GC, while the expected earnings using the simulated memory-based strategy were 48.95 GC – a non-trivial difference.

### Discussion

Experiment 1 revealed a clear cost to memory performance when task expectancy was uncertain, and suggests that participants divided attention between memory encoding and online evaluation during sequence presentation. By simulating predicted evaluation performance if people had relied on memory, we found that a pure memory-based strategy would have yielded equivalent or better performance. These findings suggest that participants adopted a suboptimal approach, incurring memory costs without corresponding benefits to evaluation.

One potential explanation for these results is that people turn list presentation into a dual-task paradigm in which memory encoding and online evaluation are mutually interfering. Compared to retrieval, divided attention at encoding typically produces substantial effects on memory performance and smaller effects on the secondary task, and the extent of impact on memory versus secondary task depends on which is given more emphasis in instructions (Craik et al., 1996) or practice (Naveh-Benjamin et al., 2006). There may have been costs to the (unobserved) latency of performing each updating step (Hogarth & Einhorn, 1992) during presentation, but no impact was observed on the accuracy of the WTP performance. While encoding and evaluation may potentially occur independently, as suggested by Hastie and Park (1986), the results confirm that the deliberate encoding required to perform on the memory task adequately could not be performed without some interference from the apparent online evaluation.

One limitation of the study is that the gathering of memory and evaluation data on different trials in the post-cued condition meant that we could not directly examine the relationship between memory and evaluation at the level of individual trials. In Experiment 2, we therefore had participants perform both tasks on each trial, allowing us to examine the relationship between memory and evaluation at the trial level, and to further examine the trade-off between memory and evaluation when both tasks are performed on each trial (cf. Mason et al., 2024).

## Experiment 2

### Method

#### Participants

We collected data from 300 participants, recruiting from Prolific and serving the study online. After exclusions as per Experiment 1, a total of 38 participants were excluded, and 262 were included in the analysis (69 in the single WTP condition, 105 in the single memory condition, and 88 in the both condition).

**Fig. 3:**
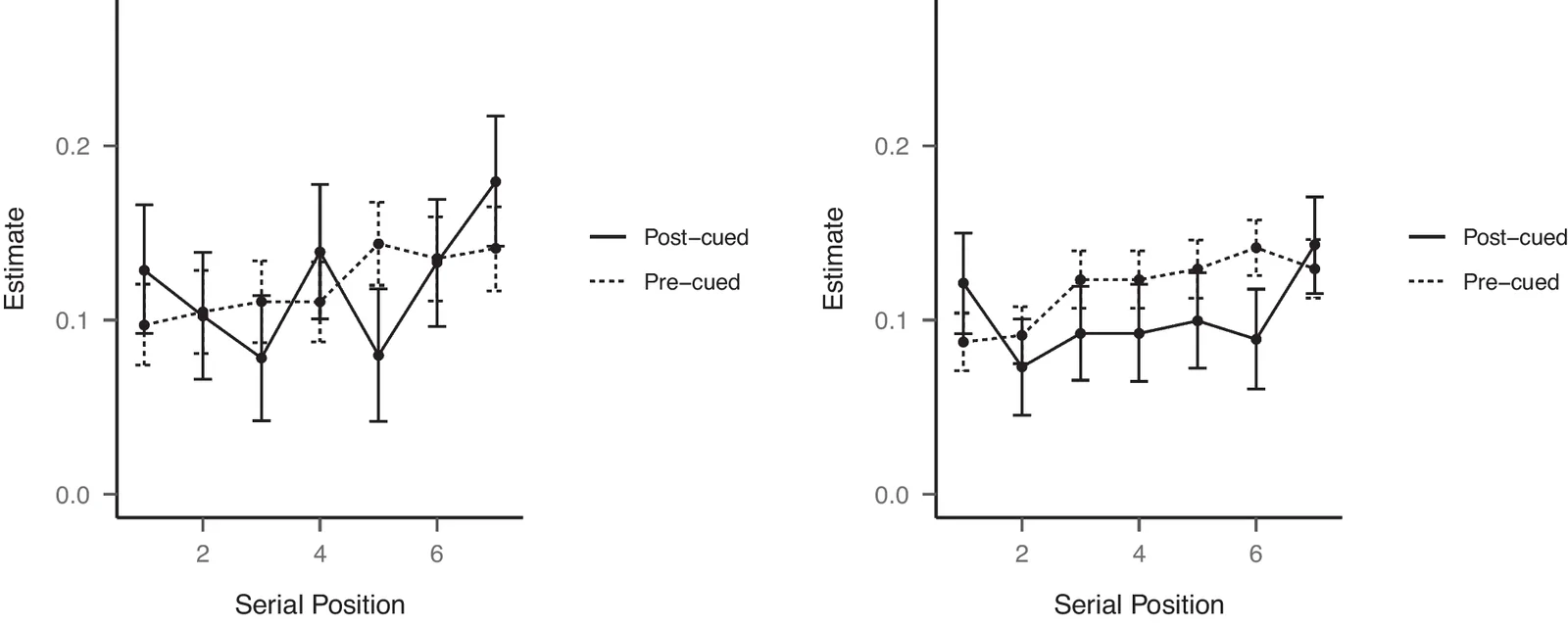
Weighting of values at each serial position in determining observed WTP, as a function of condition, for Experiment 1 (*left panel*) and Experiment 2 (*right panel*)

#### Materials and procedure

The materials and the procedure were largely the same as for Experiment 1, except that the post-cued condition required participants to perform both tasks on each trial (in an order counterbalanced across trials per participant); we refer to this as the *both* condition to make clear that both tasks were cued, and distinguish it from *single* cases were only a single task was performed. Participants in the *both* condition were incentivized for both tasks, but at half the exchange rate of those in the other conditions to approximately match the earning potential across conditions.

To equate the time demand for participants across the conditions, participants in the *single* conditions completed 32 trials, while those in the *both* condition completed 24 trials.

### Results

The right panel of Fig. 1 shows the effects of the task expectation on recall performance in the memory task. While there is a similar pattern to that seen in Experiment 1, the difference between conditions is less pronounced. The best-supported model in a Bayesian ANOVA included serial position and condition as factors, but this was only weakly preferred over a model containing only serial position (*BF*=1.13), indicating ambiguous evidence for an effect of condition. Follow-up exploratory analyses in Materials found that within the both condition there was an effect of task order, but that when the memory probe was presented first, there was little difference between the single and both conditions.

Comparison of the latencies in the WTP task in Experiment 2 suggested that participants were faster to make evaluations in the single condition (*M*=3944 ms) than in the both condition (*M*=4626 ms), but the evidence for this was weak (*BF*=1.53). There was evidence against a latency difference for the memory task (*M*=3002 ms vs. *M*=3130 ms, $BF_{10} = 0.27$). The left bars in the right panel of Fig. 2 show evaluation (WTP) performance as a function of task expectancy for Experiment 2. There was substantial evidence for an effect of task expectancy BF=42.61, with more accurate estimations in the single condition. The right two bars show performance predicted from memory performance. The “Memory (both)” shows accuracy of evaluation predicted for each participant based on their recall on the same trials, while the “Memory (single)” predicts performance in the single condition (as in Experiment 1). In both cases, performance is similar to that for participants in the single condition, again indicating that participants would not have suffered any performance loss if they had used their recall to perform evaluation. The weightings of position, calculated as in Experiment 1, show a similar pattern to that seen in Experiment 1, with more primacy and recency in the both condition (Fig. 3), but model comparison suggests no difference in the pattern of weightings between conditions (BF=11.76 favoring the model with only serial position).

The results so far suggest that Experiment 2 showed the same trade-off that was evident in Experiment 1, but with the cost being shifted from the memory task (which showed a smaller cost in Experiment 2) to the evaluation task (which showed a larger cost in Experiment 2). These costs were again found to confirm the non-optimality of the trade-off: participants in the both condition scored an average of 39.76 GC, while the expected earnings using the simulated memory-based strategy were 45.26 GC.

**Fig. 4:**
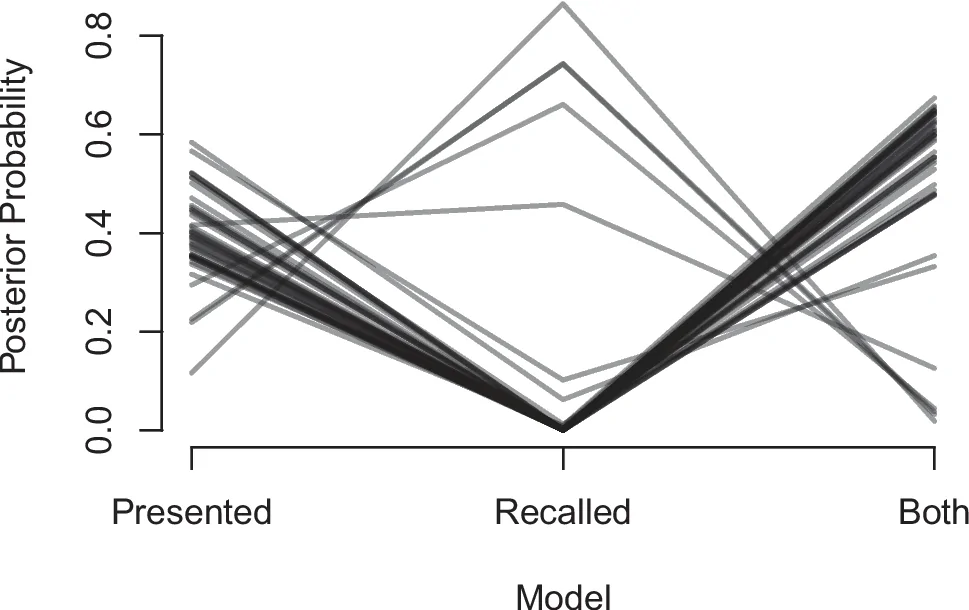
Posterior probability estimates for the model based on presented values, recalled values, or both

Given that participants in the both condition performed both tasks on each trial, we were able to examine the relationship between memory and evaluation performance at the level of individual trials. Following Mason et al. (2024), this was done by fitting a Bayesian linear mixed effects model to predict WTP on each trial from one of two predictors: the average of the presented values (as would be expected from averaging), and the average of recalled values (as would be expected from recall-based evaluation). Posterior probabilities were calculated for models using one or the other of those predictors, and a third model using both predictors. Figure 4 shows the posterior probabilities for each of those models, each line representing a single participant. The models based on presented values, or both presented and recalled values, had the highest posterior probability for most participants, with only a small number of participants showing a higher posterior probability for the model based on recalled values alone. Accordingly, most participants appeared to rely wholly or partly on online evaluation.

### Discussion

Experiment 2 replicated the trade-off between memory and evaluation seen in Experiment 1, but with a higher cost to evaluation and a smaller cost to memory. This again confirms that participants were apparently treating the task as a dual-task, with costs to one or the other task, but with no apparent benefit to either. The trial-level analysis of the both condition showed that most participants relied (at least in part) on online evaluation, with only a small number relying on recall alone (Mason et al., 2024).

Why did the cost fall primarily on memory in Experiment 1 but on evaluation in Experiment 2? One contributory factor may be that in Experiment 2, the counterbalanced task order meant that both memory and evaluation were often delayed tasks. For memory, this delay eliminated the short-term recency advantage that characterizes immediate free recall (Glanzer & Cunitz, 1966, see Fig. 1). In Experiment 1, where recall was always immediate, participants in the post-cued condition may have sacrificed some deliberate memory encoding and relied on recency to maintain an acceptable level of memory performance. In contrast, Experiment 2 participants knew any immediate recency advantage would often be lost to a filled delay, reducing the relative cost of the divided attention to encoding cf, Baddeley et al. (1969). For evaluation, however, the delay in Experiment 2 may have been particularly costly: if participants maintained a running average in working memory during presentation (Brezis et al., 2015; Hogarth & Einhorn, 1992), this summary representation would be vulnerable to forgetting during the intervening recall task. Accordingly, the shift in costs across experiments may reflect the different vulnerabilities of memory and evaluation to the effects of delay.

## General discussion

These experiments aimed to examine the potential trade-off between memory and online evaluation during presentation of an evaluative sequence, and to examine how people manage that trade-off. The results showed a cost to memory performance when participants were uncertain about whether they would be required to perform evaluation or recall, and a cost to evaluation performance when participants were required to perform both tasks on each trial. In both experiments, simulations of evaluation, predicted from memory performance, showed that had participants simply relied on memory to perform their evaluation, they would have maintained performance on both the memory and evaluation tasks without the observed cost to accuracy.

The pattern of performance across the two experiments implies that participants turned the sequence presentation into a divided attention task, with concomitant costs. In particular, in Experiment 1, the pattern is consistent with participants performing some form of online updating in the pre-cued condition, and that same process was prioritized in the pre-cued task, to the detriment of memory encoding. The Supplemental Material reports an additional memory measure, the lag conditional response probability function, which measures the tendency to make local and forward-going transitions during recall. That analysis shows that memory search is less directed in the post-cued condition (though the evidence for this effect was relatively weak); this mirrors effects of explicit concurrent task imposition at encoding (Bhatarah et al., 2006), and provides further evidence for some dividing of attention at encoding. Based on Bhatarah et al. (2006), who examined effects of concurrent load on rehearsal, one explanation is that online updating interferes with forward rehearsal of items, but could also arise from reduced binding of items to their temporal contexts or less effective updating of temporal context during encoding (e.g., Farrell, 2012; Howard et al., 2006). In Experiment 2, memory performance was largely unaffected by requiring both evaluation and recall, and the cost instead shifted to the evaluation task; we speculated that this may have arisen from participants anticipating a substantial cost to recall under a delay unless deliberate memory encoding was protected during sequence presentation.

Numerous previous studies have suggested that people optimally shift between use of different processes or strategies to maximize reward (e.g., Nicholas et al., 2022; Lengyel & Dayan, 2007; Poldrack et al., 2001; Bornstein et al., 2017; Noh et al., 2023; Brezis et al., 2015). The present results show that this is not always the case. Here, the detriment to one task was not compensated for by an increase in performance in the other task. The empirical simulation showed that had participants in the post-cued condition (Experiment 1) or the both condition (Experiment 2) focused on encoding of items (to preserve memory performance), and performed evaluation retrospectively on the basis of those items, they would have performed well on both tasks. Indeed, expected performance under that strategy exceeded that actually observed by around 12% (Experiment 2) to 25% (Experiment 1), a non-trivial cost in real terms.

How then might people choose between strategies in this context? Ludwig et al. (2025) noted that finding the optimal parameters is a substantial challenge for a participant: the objective function is unlikely to be available “laid out” in its full form for the participant to inspect, and so achieving optimal performance may require exploration of the parameter space and learning from feedback, or may rely on more heuristic approaches to satisfice on “good enough” performance. One difference between our study and other studies examining choices between different solutions is that those studies usually examine different solutions (e.g., incremental learning vs episodic memory) to the same problem (e.g., maximize rate of correct responding on a learning task). Here, the post-cued participants needed to integrate across two different tasks, and although these were put in the same currency of Galactic Credits, participants may nonetheless have treated these as incommensurable (Walasek & Brown, 2024) given they arose from different tasks, and adjudicated between them using noncompensatory meta-strategies.

One possible explanation for our results is that people may have an overarching preference for online updating, and partially relied on that even where a pure memory-based approach was more appropriate. Hoffmann and Hosch (2023) found that for shorter sequences of the type examined here, people seemed to have a preference for a more accurate averaging-based strategy over basing judgments on memory. One explanation in Mason et al. (2024) for the use of online averaging was that it arose from a more intuitive system similar to ensemble perception (e.g., Brezis et al., 2015). The results here imply that the averaging process was attentionally demanding and seems more compatible with the use of a more effortful and deliberate updating process (Hogarth & Einhorn, 1992) suggested to be used for shorter sequences presented at a slow rate (Brezis et al., 2015). This is only a partial explanation; in their experiment that post-cued memory and evaluation, Mason et al. (2024) found that some participants preferred to rely solely on memory, or a mixture of memory and online updating, implying that in many cases people prefer to base performance solely or partly on an averaging strategy, but also prefer to rely on memory; we replicated this result in Experiment 2. One general heuristic might be that people prefer to have different sources to rely on as a risk mitigation strategy in case one source is corrupted (e.g., people forget their evaluation, or happen to be unable to recall many or any instances from the sequence). Future research might seek to understand how other manipulations such as length of sequence (Brezis et al., 2015), uncertainty (e.g., Nicholas et al., 2022), and delay (Aldrovandi et al., 2015; Montgomery & Unnava, 2009) affect the balance of strategies used post-presentation, at the point of evaluation.

## Supplementary Information

Below is the link to the electronic supplementary material.

Supplementary file 1 (pdf 149 KB)

## Data Availability

The data and preregistration for the experiments are available at https://osf.io/ean6u/ and https://osf.io/cvpmy.

## Code Availability

Analysis code available at https://osf.io/cvpmy.
