# Supplementary material for: Trade-offs between remembering and online evaluation in retrospective evaluation
Source: Psychon Bull Rev. 2026 Jul 31;33(6):202. doi: 10.3758/s13423-026-02970-z (PMC13427993; doi:10.3758/s13423-026-02970-z)

Supplementary Material: Trade-offs between remembering and online evaluation in retrospective evaluation

Alice Mason[1], Geoff Ward[2], Gordon D. A. Brown[3], and Simon Farrell[4]

[1]University of Bath

[2]University of Essex

[3]University of Warwick

[4]University of Western Australia

Abstract

This document presents supplementary material for the article "Trade-offs between remembering and online evaluation in retrospective evaluation" by Mason, Ward, Brown, and Farrell. It includes examination of lag-CRP functions, and additional analyses for Experiment 2 examining ordering effects in the dual-task condition.

Keywords: memory, evaluation, dual-task, willingness-to-pay

Supplementary Material: Trade-offs between remembering and online evaluation in retrospective evaluation

Lag Conditional Response Probability Functions

Figure S1 shows the lag conditional response probability functions (lag-CRPs) for the free recall task for the pre-cued and post-cued conditions. The lag-CRP is a measure of the probability of recalling an item as a function of the lag in input position between the item just recalled, and the input position of items that could be recalled next. As typically found in free recall (e.g., Howard & Kahana, 1999), Figure S1 shows a strong "lag-recency" effect (Kahana, 1996) whereby recall of an item tends to be recalled from an item from a nearby serial position, particularly the following item at lag +1.

For Experiment 1, the figure also shows a diminishing of the lag-recency effect in the post-cued condition, consistent with effects of concurrent task performance at encoding (Bhatarah et al., 2006), though the pre-registered Bayes Factor analysis found relatively weak evidence for this effect over the model containing just lag (BF=2.95).

For Experiment 2, there is little apparent effect of condition; comparisons against the model with only the lag effect revealed evidence against an effect of condition (BF=5.56 favouring the reduced model) and against an interaction between condition and lag (BF=12.43 favouring the reduced model).

Consideration of ordering effects in the Both condition in Experiment 2

Figure S2 plots recall accuracy in the free recall task as a function of the order in which the free recall and willingness-to-pay tasks were performed in the Both condition of Experiment 2, alongside memory performance in the Single condition. A mixed ANOVA focussing on the Single condition versus the Both condition when the memory task was first found that the best-fitting model included only an effect of serial position; this model was supported over the next best-fitting model that also included the effect of Both vs Single (BF=2.86) indicating evidence against that factor. A repeated-measures ANOVA examining ordering effects within the Both condition found that the best-fitting model

included effects of both task order and the interaction; this model was only weakly supported over a model including the serial position factor as well (BF=2.56) but was strongly supported over the null model (BF=59.98). Figure S2 and the analyses imply that delaying recall by interpolating the willingness-to-pay task had a detrimental effect on recall accuracy, and that when restricting attention to cases where the recall task immediately followed presentation, there was little apparent difference between the Single and Both conditions.

Figure S3 plots willingness-to-pay evaluation accuracy as a function of the order in which the free recall and willingness-to-pay tasks were performed in the Both condition of Experiment 2, alongside evaluation performance in the Single condition. The figure shows that the ordering of the tasks had a non-detectable effect on evaluation accuracy, or on the difference with respect to the Single condition.

Bias and variance in estimation

The main paper reports an aggregate RMSD score of evaluation accuracy. Here, at the suggestion of a reviewer, we decompose this into measures of bias and variance in estimation. Because the "true" value varied from trial to trial, we cannot calculate bias and variability directly on the values. Instead, we use linear regression to estimate bias as the intercept in a regression of the estimated value on the true value, and variability as the standard deviation of the residuals from this regression. The regression was performed using brms (Bürkner, 2017) in R, with random variability in intercept across participants.

In Experiment 1, in the pre-cued condition the mean intercept was -.11 [95% credible interval: -3.02, 2.95], and the estimated standard deviation of the residuals was 7.90 [95% credible interval: 7.60, 8.22]. In the post-cued condition, the mean intercept was 0.14 [95% credible interval: -1.93, 2.19], and the estimated standard deviation of the residuals was 8.27 [95% credible interval: 7.81, 8.76]. Thus there was no indication of a bias in estimates, or a trade-off between bias and variance across conditions.

In Experiment 2, in the single condition the mean intercept was 1.66 [95% credible

interval: -0.22, 3.69], and the mean standard deviation of the residuals was 8.36 [95% credible interval: 8.15, 8.57]. In the both condition the mean intercept was -2.51 [95% credible interval: -4.98, -0.21], and the mean standard deviation of the residuals was 11.28 [95% credible interval: 10.94, 11.66].These results suggest that the dual-task manipulation produced a small shift in bias between conditions, and a reliable increase in residual variability.

Figure S1

Lag conditional response probability functions for free recall

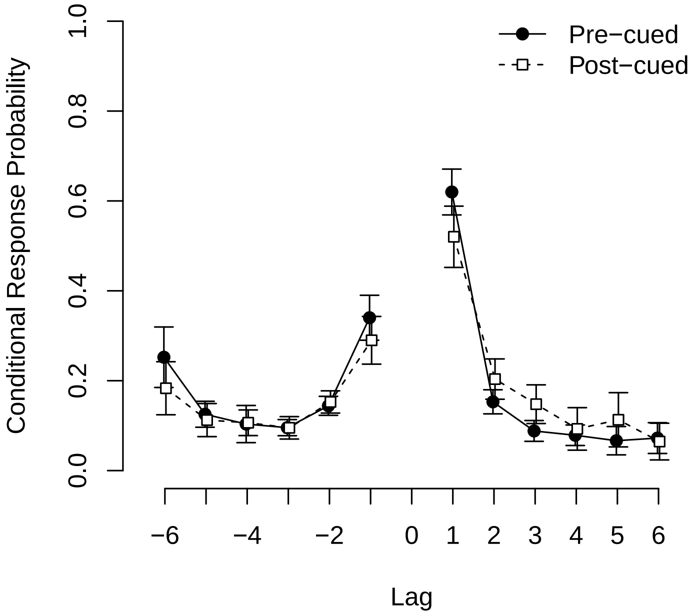

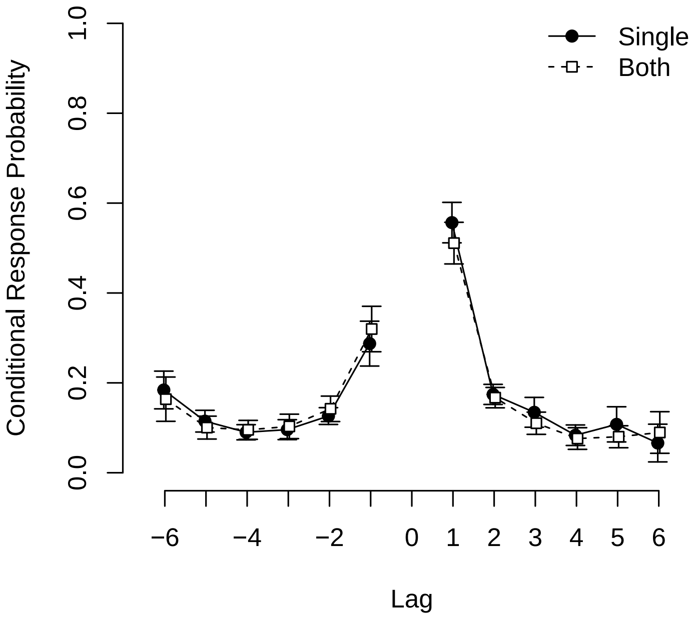

Figure S2

Effects of task order on recall accuracy in Experiment 2

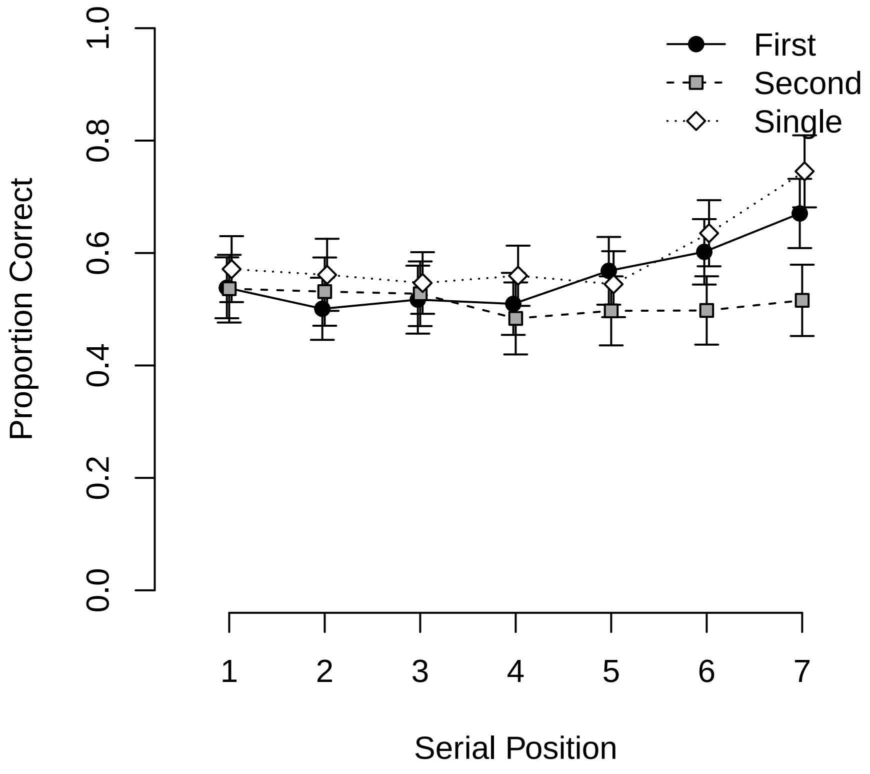

Figure S3

Effects of task order on evaluation accuracy in Experiment 2

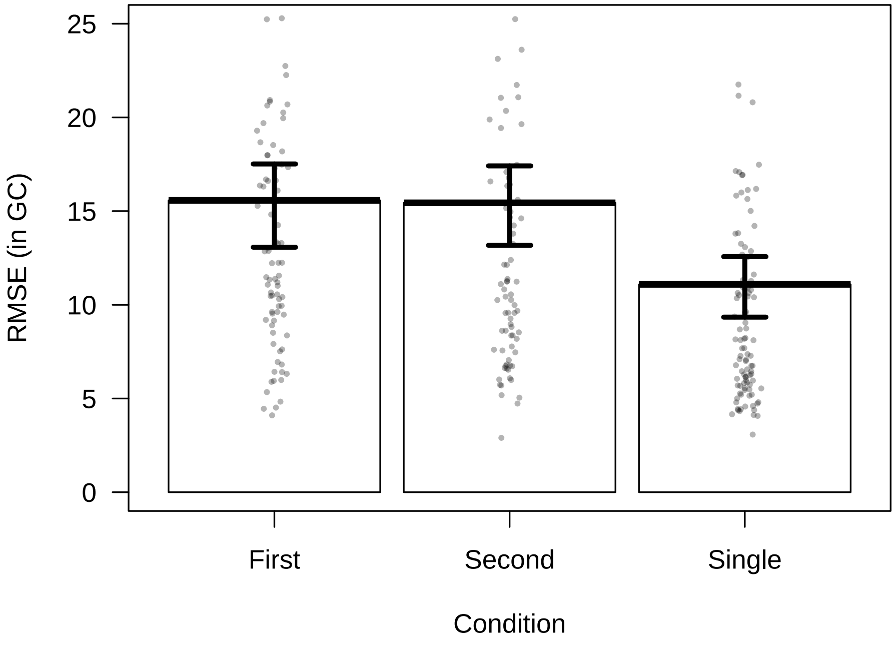

Supplement: Supplementary file 1 — (pdf 149 KB) [file 13423_2026_2970_MOESM1_ESM.pdf]
